# Supplementary material for: Chasing the Apomictic Factors in the Ranunculus auricomus Complex: Exploring Gene Expression Patterns in Microdissected Sexual and Apomictic Ovules
Source: Genes (Basel). 2020 Jun 30;11(7):728. doi: 10.3390/genes11070728 (PMC7397075; doi:10.3390/genes11070728)
Supplement: Supplementary file 1 [file genes-11-00728-s001.zip › Figure S2. STEM_V2.docx]

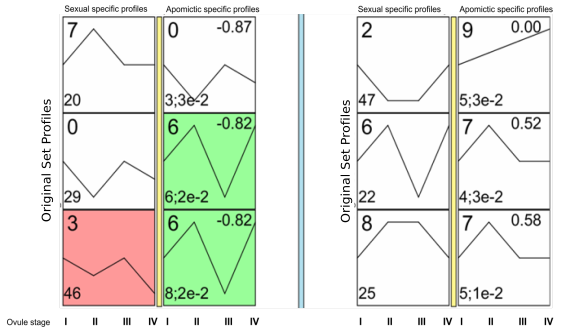


**Figure S2.** Analysis of significant intersection between model profiles from sexual (original set profiles; left of the yellow line) and apomictic (comparison set profiles; right of the yellow line) gene expression throughout ovule development derived from STEM (Ernst and Bar-Joseph, 2006). In each comparison, differentially expressed genes in the intersection of model profiles from the sexual and apomictic libraries were identified. For each profile, the pattern of expression across 4 developmental stages (plotted equally along the x-axis) is plotted using log2 normalized data (each y-axis tick represents two log2 intervals of normalized gene expression). Each model profile is anchored at the 0 for the first time point, and colored plots demonstrate model profiles for which there were an excess of assigned versus expected Differentially expressed genes (P<0.01) (Ernst and Bar-Joseph, 2006). For each box up left number represent assigned profile number, lower left number represent the number of genes for that particular pattern of expression. For boxes left of the yellow line, lower left number represent the number of common genes (in respect to the sexual profile ) genes assigned to a different pattern and the p value associated, the upper right number che correlation between the 2 profiles (Ernst and Bar-Joseph, 2006).
