# Supplementary material for: Chasing the Apomictic Factors in the Ranunculus auricomus Complex: Exploring Gene Expression Patterns in Microdissected Sexual and Apomictic Ovules
Source: Genes (Basel). 2020 Jun 30;11(7):728. doi: 10.3390/genes11070728 (PMC7397075; doi:10.3390/genes11070728)
Supplement: Supplementary file 1 [file genes-11-00728-s001.zip › Figure S3. Expression pattern changes DEG.docx]

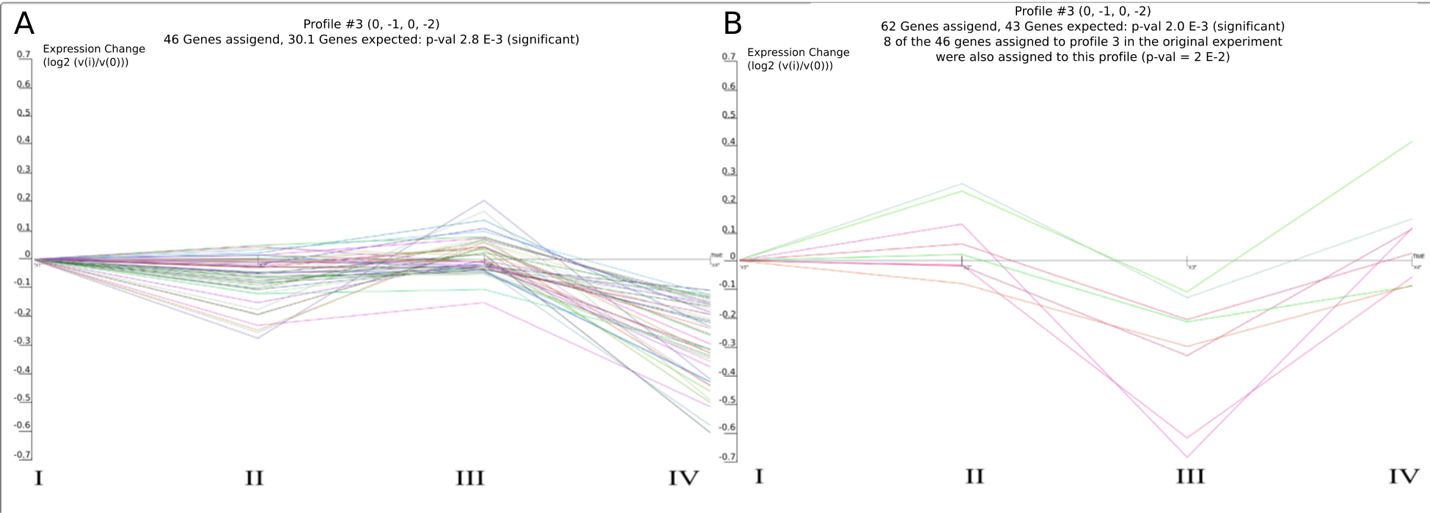


**Figure S3.** Graph showing expression pattern of genes in sexual specific ovule development (A) and the homologous genes expression pattern in apomictic ovule development (B) with statistically significant heterochronic expression according to STEM software (Ernst and Bar-Joseph, 2006). The roman numbers on the x-axis represent the 4 ovule developmental stages, the y-axis the log2 intervals of normalized gene expression changes (Ernst and Bar-Joseph, 2006).
