# Supplementary material for: Chasing the Apomictic Factors in the Ranunculus auricomus Complex: Exploring Gene Expression Patterns in Microdissected Sexual and Apomictic Ovules
Source: Genes (Basel). 2020 Jun 30;11(7):728. doi: 10.3390/genes11070728 (PMC7397075; doi:10.3390/genes11070728)
Supplement: Supplementary file 1 [file genes-11-00728-s001.zip › Table S3. Genes showing similar 2x-6x expression pattern and 4x parent of origin effects.docx]

**Table S3.** Genes associated with each ovule developmental stage that showed similar expression between hexaploid apomicts and diploid sexuals but were differentially expressed in the tetraploid.

| **Expression class ^1^** | **Develop. stage** | **Transgressive effect** | **Parent of Origin effect** | **Ploidy effect** | **Total** |
| --- | --- | --- | --- | --- | --- |
| 6x apomictic/2x sexual **overexpressed** | I | - | 2750 | - | 2750 |
|  | II | - | 2874 | - | 2874 |
|  | III | - | 2909 | - | 2909 |
|  | IV | - | 2551 | - | 2551 |
|  | Total | - | 11084 | - | 11084 |
| 6x apomictic/2x sexual **underexpressed** | I | - | 4630 | - | 4630 |
|  | II | - | 4887 | - | 4887 |
|  | III | - | 5168 | - | 5168 |
|  | IV | - | 4395 | - | 4395 |
|  | Total | - | 19080 |  | 19080 |
