# Supplementary material for: Chasing the Apomictic Factors in the Ranunculus auricomus Complex: Exploring Gene Expression Patterns in Microdissected Sexual and Apomictic Ovules
Source: Genes (Basel). 2020 Jun 30;11(7):728. doi: 10.3390/genes11070728 (PMC7397075; doi:10.3390/genes11070728)
Supplement: Supplementary file 1 [file genes-11-00728-s001.zip › Table S6. List of genes and primers used for microarray validation.docx]

**Table S4.** List of genes used for qRT-PCR validation.

In red and green transcript up and down regulated , respectively (according to the microarray analysis)

| **Seq id*** | **Primer 5´- 3´** | **primer 3´-5´** | **Expression** | **P Value** | **Result** |  |
| --- | --- | --- | --- | --- | --- | --- |
| 345733 | ACCAAAGCGATTAGAAACACCAGT | CCCGAGAAAGCCGATGAAA | 0.467 | 0.089 | Up Regulated |  |
| 350109 | TCTTGATTCGATTCTTGTGGTTGA | TGTTCCGGTGGCGACTTG | 131.622 | 0 | Up Regulated | |
| 354538 | CAGGCGCAAATGTGAGA | TGGGATCGATAGCAAGAGTC | 36.07 | 0.009 | Up Regulated | |
| 357979 | GGCTCTGAGTCAACTTGTCCTAAT | CTTTCGGCTTCCTTTTCTTCTT | 4.286 | 0 | Up Regulated | |
| 360024 | CGACGAGACGGGGTTGAA | ATTGGCGTTGCATCTCTAAGTG | 34.088 | 0.014 | Up Regulated | |
| 366262 | ACGTGGTGTTTGGGAATGAAT | TGAACGGATATCCCTGACAAAA | 3.772 | 0 | Up Regulated | |
| 411537 | GAGCCGGAAAGTGCGAGTA | GATGCTGTGATGGAATTGATAGTA | 0.018 | 0.023 | Down Regulated | |
| 505317 | GGAAATCCAAGAAACAACC | GAAGATCATTAAGCAAGTGG | 0.24 | 0.057 | Down Regulated | |
| 556851 | GATTTTAGGCCATTTGATTGTGC | TTGAGGGTTTGTATGAGATTTGAA | 0.006 | 0.02 | Down Regulated | |
| 345754 | TAGCCAACGTACCGATTAGGATAG | AGGTAGTGGCTTGCTCTTTCTGTC | 0.033 | 0.016 | Down Regulated | |
| 405990 | ACCCCAGCTGTCTTCCGAGTA | CACAAATGGCATATGAGACACG | 0.251 | 0.031 | Down Regulated | |

* Dryad entry doi:10.5061/dryad.nk151;
