# Supplementary figures and images for: Chasing the Apomictic Factors in the Ranunculus auricomus Complex: Exploring Gene Expression Patterns in Microdissected Sexual and Apomictic Ovules
Source: Genes (Basel). 2020 Jun 30;11(7):728. doi: 10.3390/genes11070728 (PMC7397075; doi:10.3390/genes11070728)

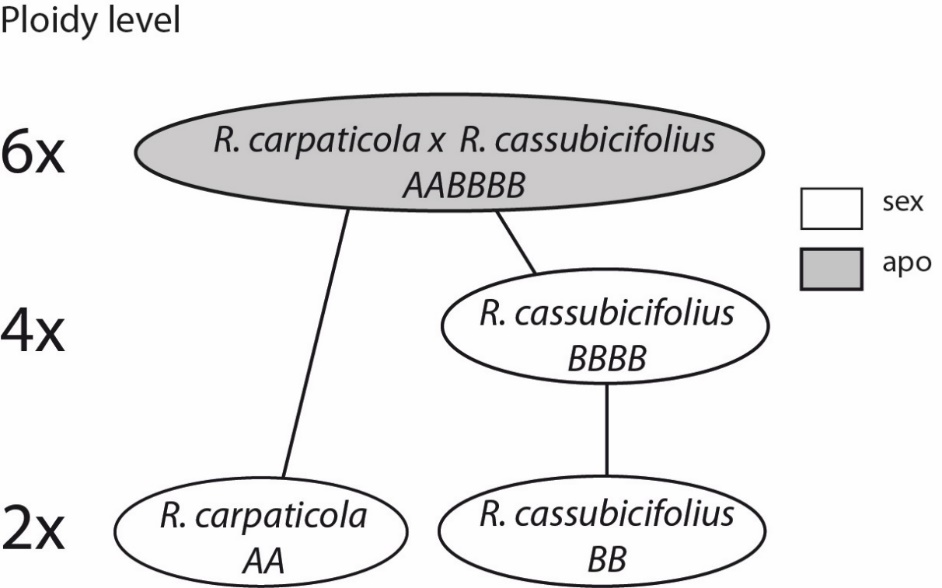


**Figure S1.** Relationships and genome contributions of the taxa

Supplement: Supplementary file 1 [file genes-11-00728-s001.zip › Figure S1. Relationships and genome contributions of the taxa.docx]

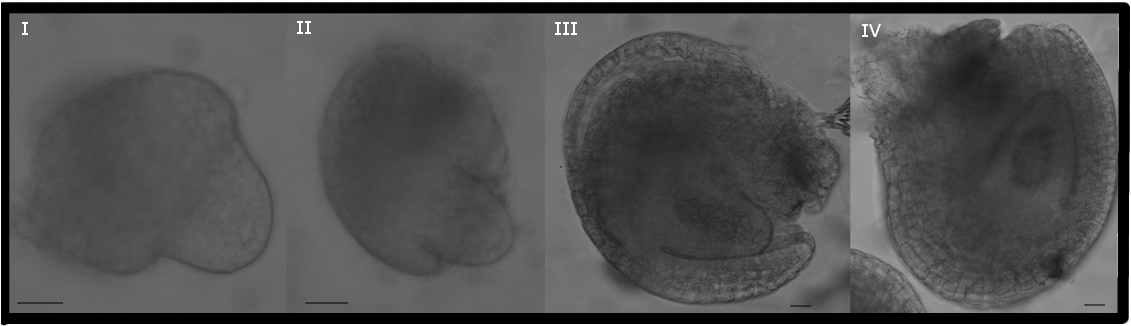

Supplement: Supplementary file 1 [file genes-11-00728-s001.zip › Figure S4. Example of Ovule staging.tif]
